# Short-Term Forest Management Effects on a Long-Lived Ectotherm

**DOI:** 10.1371/journal.pone.0040473

**Published:** 2012-07-06

**Authors:** Andrea F. Currylow, Brian J. MacGowan, Rod N. Williams

**Affiliations:** Department of Forestry and Natural Resources, Purdue University, West Lafayette, Indiana, United States of America; Hungarian Natural History Museum and Eotvos University, Hungary

## Abstract

Timber harvesting has been shown to have both positive and negative effects on forest dwelling species. We examined the immediate effects of timber harvests (clearcuts and group selection openings) on ectotherm behavior, using the eastern box turtle as a model. We monitored the movement and thermal ecology of 50 adult box turtles using radiotelemetry from May–October for two years prior to, and two years following scheduled timber harvests in the Central Hardwoods Region of the U.S. Annual home ranges (7.45 ha, 100% MCP) did not differ in any year or in response to timber harvests, but were 33% larger than previous estimates (range 0.47–187.67 ha). Distance of daily movements decreased post-harvest (from 22 m±1.2 m to 15 m±0.9 m) whereas thermal optima increased (from 23±1°C to 25±1°C). Microclimatic conditions varied by habitat type, but monthly average temperatures were warmer in harvested areas by as much as 13°C. Animals that used harvest openings were exposed to extreme monthly average temperatures (∼40°C). As a result, the animals made shorter and more frequent movements in and out of the harvest areas while maintaining 9% higher body temperatures. This experimental design coupled with radiotelemetry and behavioral observation of a wild ectotherm population prior to and in response to anthropogenic habitat alteration is the first of its kind. Our results indicate that even in a relatively contiguous forested landscape with small-scale timber harvests, there are local effects on the thermal ecology of ectotherms. Ultimately, the results of this research can benefit the conservation and management of temperature-dependent species by informing effects of timber management across landscapes amid changing climates.

## Introduction

Study of habitat alteration through direct and indirect anthropogenic episodes such as reduction of forest habitats and changing climate is becoming increasingly frequent. The understanding of how these changes affect the physiology and behavior of native fauna is vital to the preservation of diversity. Timber harvesting is likely one of the most prominent land uses affecting forest wildlife [1]–[5]. Forest management practices change the vegetative structure and local temperature, which may affect community structure and function [6]. Environmental flux also has a greater effect on movements and behavior of poikilotherms than for homeothermic species [7], [8]. In response, timber harvests have been implicated as a possible cause for worldwide herpetofaunal declines [9]–[11]. As a result, management of our eastern hardwood forests has become a balancing act between timber production and ecological conservation.

While some data suggest that heavily logged areas are associated with moderate increases in bird and reptile diversity [3], it is not clear whether this can be considered a general trend for all taxa. Timber harvesting has the potential to affect multiple facets of how ectotherms utilize available habitat both directly and indirectly. Canopy openings may create basking sites or allow herbaceous mass to flourish and provide basilar food sources [12]. Edge effects of openings and access roads have been shown to influence habitat resources into the forest interior at varying distances [13]–[15]. Because variation in resources such as vegetation and invertebrate prey occur, daily movements and annual home range sizes may readily expand, contract, or shift in response to this variation. Moreover, the behavior, physiology, and even fitness of ectotherms are strongly affected by temperature fluctuations [16], [17]. Temperature dictates ectothermic habitat use based on the animals thermal optima (i.e., the temperature at which movement activity is maximal; [17] which in turn alters behavior [18], [19].

Recent attempts to assess the effects of timber harvests on many ectothermic species often suffer from the lack of replication or comparable pre-harvest data (e.g., [20], [21]). Furthermore, the majority of these herpetofaunal studies have focused on the harvest effects on amphibian populations (e.g., [5], [22]–[25]), while relatively little is known about the impacts on reptile populations. However, the existing data suggest reptiles are not only sensitive to habitat perturbations, but that the impacts are more pervasive and severe than for amphibians [11]. Negative impacts to reproductive adult reptiles, such as long-lived, K-selected turtles, can devastate entire populations [26], [27]. Box turtles, which are among the longest lived of all reptiles, are geographically widespread throughout the eastern forests, yet they are sensitive to environmental disturbances that affect local habitat features [28]–[30]. Widespread population declines have sparked interest in the conservation of this species. While basic data exist on the habitat requirements of certain ectothermic species, many studies were conducted at a single location and did not empirically assess responses to changing habitat or microenvironmental conditions.

The investigation of ecological mechanisms underlying species declines has become paramount in conservation literature. Simply reporting the extirpation of populations without testing mechanistic causes does little to promote conservation management. Herein, we investigated temporal thermal habitat availability, habitat use, thermal behavior, and intersexual differences among eastern box turtles (*Terrapene carolina carolina*) within the framework of a managed forest setting. The overarching goals of this study were to examine ectothermic response to timber harvesting at both the landscape- and local-scale. At the landscape scale, our specific goals were to assess effects of various timber harvest regimes on habitat use, thermal environments, and thermal ecology. At the local level, our specific goals were to investigate edge effects of timber harvests on thermoregulatory behavior, movement parameters (frequency of movement and steplength), and observed behavior.

## Methods

### Study area

**Figure 1:**
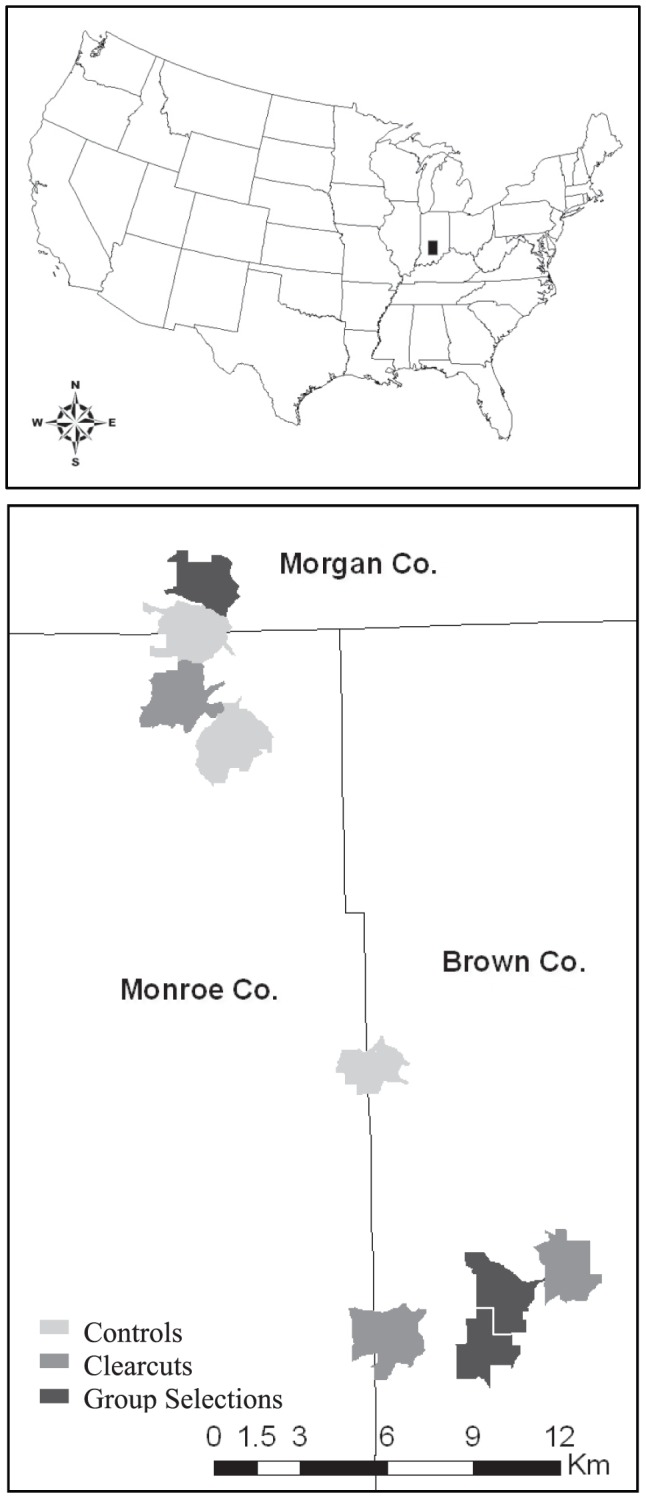
Study Area Maps. Regional and local map of the study area in south-central Indiana. a) The location of the study area in Indiana relative to the continental US. b) The nine study sites spanning Morgan, Monroe, and Brown Counties in IN. Polygon colors represent management classes (controls = light grey, clearcuts = medium grey, group selections = dark grey).

The research was conducted within approximately 35,000 hectares of Morgan-Monroe State Forest (MMSF) and Yellowwood State Forest (YSF) in Morgan, Monroe, and Brown Counties, Indiana (Figure 1a). From the years 1860 through 1910, routine burning and cutting for cattle grazing characterized the forestland. At the turn of the 20^th^ century, the state of Indiana began purchasing the land and establishing these State Forests. Now, MMSF and YSF boundaries are shared, forming a relatively contiguous forested habitat characterized by hills and ravines of hardwood, deciduous forests with scattered gravel access roads. This is an oak-hickory dominated forest, with the majority of canopy species being *Quercus* spp., such as *Q. montnana* (chestnut oak), and *Carya cordiformis* and *C. ovata* (butternut and shagbark hickory; [31]). These State Forests are managed for multiple-uses, including recreation, education, research, and timber harvesting. Research activities on public lands were conducted under the scientific use permits 09-0080 & 10-0083 issued by the Indiana Department of Natural Resources.

### Forest management design and sampling

Our research is part of a long-term (100-yr), landscape-scale (spanning 31 linear kilometers and 3,601 hectares) timber and wildlife research collaborative designed for the study of ecological and social impacts of various silvicultural methods typically employed in the Midwest (Hardwood Ecosystem Experiment [32]. In 2007, we identified nine study sites of approximately 400-ha, each assigned to one of three forest management classes in a randomized complete block design (Figure 1b). The management classes included two 2.72–4.43-ha clearcuts, eight 0.15–2.55-ha group selection openings, and forested controls. The timber harvests were implemented on equal numbers of southwest- and northeast-facing slopes over the winter of 2008–09 within the center 90-ha of each study site. The remaining 300+ hectares at each site remained intact to serve as refugia and maintain species diversity.

To determine the effects of timber harvests on *T. c. carolina*, we collected GPS location and habitat use data before timber harvests (pre-harvest; 2007–08) and after harvests (post-harvest; 2009–10). We initially located adult animals by meandering-transect visual encounter surveys. Upon capture, we assigned a unique ID number and marked each animal using a triangle file along the marginal scutes following a modified Cagle scheme [33]–[35], recorded morphometrics, and affixed a transmitter (model RI-2B Holohil Systems, Ltd., Ontario, Canada) to the carapace. Where possible, we equally divided sex ratios and numbers of the animals among sites and management classes. We subsequently radio-tracked (homing) the animals 2–3 times per week during the active seasons (May through October). For each tracked location, we recorded GPS coordinates, date, ground temperature, elevation, and during the post-harvest years we also recorded observed activity classifications (resting, eating, mating, basking, walking, etc.).

To monitor the thermoregulatory behavior of the animals post-harvest, we affixed iButton temperature dataloggers (model DS1921G-F5#, Maxim Integrated Products, Inc., Sunnyvale, CA) to the carapace of each of the tracked turtles in May 2009. Since carapacial temperature measurements have been shown to correlate well with deep body temperatures [36]–[39], we used the dataloggers to represent each animal's body temperature (T_b_). Temperature datalogger and transmitter weight combined was usually no more than 5% (max 20 g) of the animal's total body weight. Dataloggers recorded temperatures every 45 minutes during the active season (May–October). All animals were handled according to the Purdue Animal Care and Use Protocol 07-037.

To assess the available thermal habitats in harvest areas versus uncut forests, we measured ambient temperature using temperature dataloggers affixed to stakes, 10 cm from soil surface (at approximately *T. c. carolina* carapace height). We randomly placed these ‘environmental dataloggers’ at four sample locations within each of the nine study sites for a total of 36 individual thermal locations. In each clearcut management site, two environmental dataloggers were randomly deployed inside clearcuts and two in the adjacent forests (between 100 m and 500 m from the nearest harvest edge; harvest-adjacent forest). In each group selection management site, four dataloggers were randomly deployed inside harvest openings. In each control site, we randomly deployed four dataloggers within the forested habitats. This blocked design resulted in equal numbers of environmental dataloggers inside harvest openings (n = 18) and in forested areas (n = 18) representing the four habitat types (clearcut opening, group selection opening, harvest-adjacent forest, and forested control). To eliminate the effect of slope aspect on temperature logged, we used equal numbers of southwest- and northeast -facing slopes. We deployed all temperature loggers from May 2009 to October 2010 for a total of 75 weeks. We programmed dataloggers to record temperatures every 45 minutes to match the carapacial dataloggers described above.

### Landscape-scale analyses

Home Range Estimation. We used multiple analyses to examine how various timber-harvesting regimes affect behavior at landscape- and local-scales. To describe landscape-scale effects of timber harvests, we used all animal location data across all nine study sites throughout the forested landscape. To characterize spatial land use in our population of box turtles, we created a point layer in ArcGIS 9 (version 9.3.1; [40]) using the GPS location data and calculated 100% Minimum Convex Polygons (MCP) with the Hawth's Analysis Tools extension [41] for each turtle in each year, thus creating annual MCP home ranges. We standardized all annual MCP home ranges by the number of GPS locations and log-transformed them for normality.

We used a generalized linear mixed model to test annual MCP home ranges for differences among sites using a crossover design and the PROC GLMMIX command in SAS [42] with a first-order autoregressive covariance structure. We compared all the pre-harvest data then “crossed over” to the post-harvest control comparisons. In our initial model, site, year, and the interaction of site and year were fixed effects and animal ID nested in site was a random effect. By analyzing data in this crossover fashion, we could verify that control sites were representative of pre-harvest conditions (i.e., site explained very little variation). We grouped sites by management class (clearcut, group selection, and control) for all subsequent analyses and evaluated their effects in the pre- and post-harvest data using a full factorial generalized linear mixed model (GLMM) with unbounded variance components in JMP [43]. We used year, sex, management class, and their interactions as fixed effects and animal ID nested in year as a random effect (to account for repeated measures of individual animals) to find any differences in annual MCP home ranges with relation to harvests. To detect significant differences across effect levels, we used post-hoc Least Squares Means (LSMeans) Tukey-Kramer pairwise comparisons, which adjust significance for multiple comparisons.

Year-to-year variation in movements and habitat use is common (often due to variation in resources such as vegetation and invertebrate prey; [28], [44], [45], therefore we used biennial (two-year) intervals as indices of longer-term home range sizes and core use areas. These biennial intervals corresponded to the two pre-harvest years and two post-harvest years (hereafter “harvest periods”). To assess differential habitat utilization due to timber harvests, we used biennial MCPs and kernel estimates (ArcGIS Home Range Tools [HRT] extension; [46]) for each animal between harvest periods. We chose to use kernel estimates for further comparisons to other habitat use studies [47] but also continued to calculate MCPs because it has been argued that they better represent herpetofaunal habitat use [48]. We calculated 50%, 90%, and 95% kernel isopleths (percent volume contour) of utilization distributions using the fixed kernel method with least squares cross validation (LSCV) for pre- and post-harvest. For both biennial MCP and kernels, we used a GLMM to test for differences in pre- and post-harvest area measurements (log-transformed) caused by the fixed effect of harvest period (with animal ID nested as a random effect to control for re-sampling error).

### Movement and thermal ecology

Animals may not only adjust their annual home ranges in response to harvests, but also vary their movement activity (i.e. move farther distances within their home range or move more frequently). For this analysis, we calculated the Euclidian distance between GPS locations for each animal in ArcGIS using the HRT extension then calculated steplength (average estimated distances by day). To test whether harvest period had an effect on steplength, we log-transformed these data and fitted a full factorial unbounded GLMM with harvest period, sex, management class, and their interactions as fixed effects and animal ID nested in harvest period as a random effect. Then to examine the thermal ecology of *T. c. carolina* in relation to the timber harvests, we tested for correlation between the log-transformed steplength data and ground temperature (T_g_; recorded when animals were radio-located). We also used these data to determine the thermal optima (the temperature at which movement activity is maximal) across harvest periods.

### Thermal habitats

To test for changes in available thermal habitat, we used differences in ambient temperature among habitat types within sites. We summarized the temperature time series data from each of the 36 environmental dataloggers into three variables; monthly temperature maxima (T_max_), monthly temperature minima (T_min_), and monthly temperature mean (T_mean_) using R [49]. We used unbounded GLMM in JMP to test for significant T_min_, T_max_, and T_mean_ differences caused by habitat type, month, and their interaction as fixed effects and individual datalogger ID nested in month as the random effect. We used LSMeans Tukey-Kramer post-hoc comparisons to detect significant differences in T_min_, T_max_, and T_mean_ between months.

### Local-scale analyses

To determine the thermal effects of harvests on ectotherm behavior, we first characterized the thermoregulatory behavior of our entire turtle population. We examined the max, mean, and min T_b_ to find the range of selected temperatures for each month. We correlated observed behavior at the time of each GPS location in relation to T_b_. We used a GLMM to investigate animal body temperature (T_b_) differences explained by the fixed effect of observed behavior category with the random effect of animal ID nested in behavior. Behavior categories included basking, eating, mating, resting, inverted (found upside-down), walking, and buried.

To investigate local-scale harvest edge use and activity, we examined the actual harvest openings and their associated edges in combination with GPS location data. We then created 10- and 50-meter polygon buffers around the harvest boundaries using ArcGIS Analysis Tools. We tested for differences in the percent of animal locations within these three harvest-polygons (inside harvest, 10 m buffer, and within the 50 m buffer) across harvest periods, again controlling for individual effects using an unbounded GLMM as described above. We conducted a similar analysis using the Euclidian distances animals moved within these harvest-polygons to test for differences in activity (frequency of movement or daily distance moved).

To determine the edge effects on thermoregulation, we compared T_b_ of the animals using the harvests and their edges to the T_b_ of those same animals when they were located in the forests. To investigate edge effects on movement activity, we used T_b_ to describe the available thermal habitats in various harvest-polygons. We analyzed harvest edge effects by categorizing harvest proximity polygons (as above) by inside the harvest, 10 m buffer, and 50 m buffer from the nearest harvest opening. We also explored T_b_ within harvest-polygons by using T_b_ as the response variable and distance to harvest and month as the fixed effects. We used unbounded GLMMs and controlled for repeated measures using animal ID nested in harvest-polygons as a random effect in each model. We performed post-hoc LSMeans Tukey-Kramer pairwise comparisons to detect significant differences.

## Results

### Landscape-scale effects

We radio-tracked 23–44 *T. c. carolina* each year (average = 33.5/year), carrying over all that survived each year and were not lost or censored. Losses due to transmitter failure were rare (n = 1). Two animals were separated from their transmitters and censored. Five animals died of various causes including predation (n = 1), severe emaciation (n = 1), suspected disease (n = 2), or failure to emerge from hibernacula (n = 1). Home range MCPs for the remaining animals (n = 50; 23♂, 27♀) with >20 locations per year (avg. = 57.34, SD = 19.10, range = 14–79) were calculated for each year (see supporting information for Table S1).

We found no difference (*p*-value = 0.418) in the overall size of *T. c. carolina* annual MCP home ranges between all pre-harvest sites and control post-harvest sites, verifying our experiment used true controls. Annual MCP home ranges (4.10 ha to 11.43 ha) did not differ among sex, year (2007–10), management class, or any combination of these factors (all *p*-values>0.07). The minimum and maximum annual home range sizes were 0.47 ha and 187.67 ha, respectively. The average MCP for all four years was 9.14 ha for males and 5.55 ha for females.

Average pre-harvest biennial MCP home ranges (18.93 ha, SE = 7.51) were generally larger than post-harvest (9.09 ha SE = 5.75; Table 1), however, this difference was not significant (*F*_1, 2.435_ = 0.018, *p* = 0.90). There was much variation in kernel areas by sex and harvest period (Table 1) For all three kernel isopleths (50%, 90%, and 95%), the home range areas increased from pre-harvest to post-harvest (all *p*-values<0.05). No variation in biennial home range area was attributed to harvest type (clearcut or group selection) or sex (all *p*-values>0.29).

**Table 1:** Pre-harvest (Pre-harv.; 2007–2008) and post-harvest (Post-harv.; 2009–2010) home ranges of female and male eastern box turtles.

Period	Sex	Mngmnt Class*	*n*	Biennial MCP†			Biennial 95% Kernel‡		
				*Median*	*Mean*	*SE*	*Median*	*Mean*	*SE*
Pre-harv.	F	Clearcut	5	6.80	15.42	10.20	3.57	32.32	28.97
		Control	4	3.52	4.54	1.86	3.32	3.37	0.73
		GroupSelect	2	10.21	10.21	6.74	4.31	4.31	0.38
	M	Clearcut	5	2.02	4.63	1.75	2.71	4.34	1.07
		Control	4	5.52	83.08	78.41	5.39	14.99	10.63
		GroupSelect	7	3.53	5.70	2.81	3.85	4.35	1.11
Summary	F	All	11	5.27	10.52	4.74	3.94	16.70	13.15
	M	All	16	3.57	24.71	19.62	4.12	7.01	2.72
*Totals*			*27*	*3.61*	*18.93*	*11.70*	*3.94*	*10.96*	*5.52*
Post-harv.	F	Clearcut	7	2.57	10.56	5.80	1.45	1.85	0.51
		Control	8	7.96	9.87	2.81	2.28	5.02	2.91
		GroupSelect	9	2.69	5.48	1.89	1.30	1.36	0.23
	M	Clearcut	7	5.98	11.11	6.30	2.22	49.22	46.96
		Control	7	3.65	16.72	13.10	1.59	18.66	17.29
		GroupSelect	8	2.32	2.64	0.51	1.66	1.64	0.21
Summary	F	All	24	4.19	8.42	2.02	1.49	2.72	1.00
	M	All	22	3.02	9.82	4.57	1.75	22.19	15.69
*Totals*			*46*	*3.02*	*9.09*	*2.40*	*1.57*	*12.03*	*7.57*

* The associated management class (Mngmnt Class).

† biennial Minimum Convex Polygons (MCP) home ranges.

‡ Only the 95% kernel isopleths areas are listed here, as they are the only relevant comparisons to 100% MCP.

### Movements and thermal ecology

**Figure 2:**
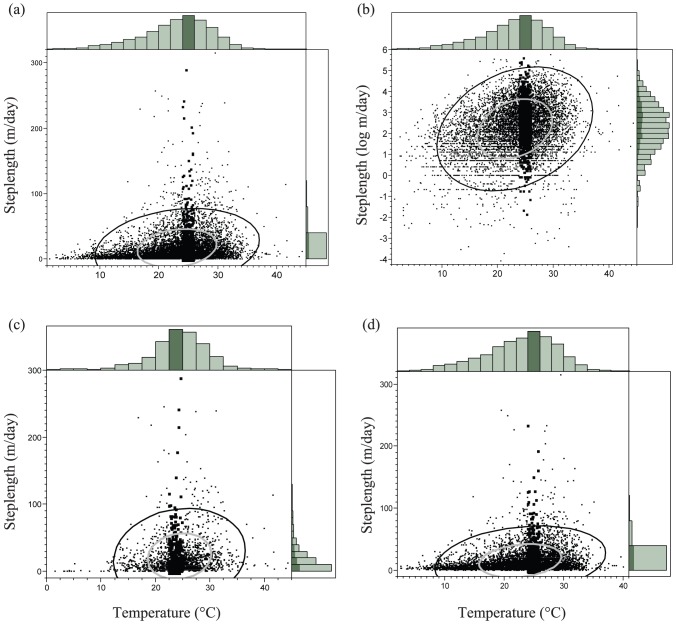
Thermal Optima. Scatter plot of daily distances traveled by eastern box turtles (steplengths; y-axes) by ground temperature (T_g_ in °C; x-axes). All 2007–10 steplengths (in meters per day) by ground temperature (a.) and the log-transformed steplength by ground temperature (b.). Pre-harvest (2007–08) steplength in meters per day by ground temperature (c.) and post-harvest (2009–10; d.). Plots show 95% (black ellipse) and 50% (grey ellipse) density ellipses around points and histogram densities along plot boarders. Darkened areas represent the peak of activity temperatures (22–26°C; thermal optimum) in these data.

**Figure 3:**
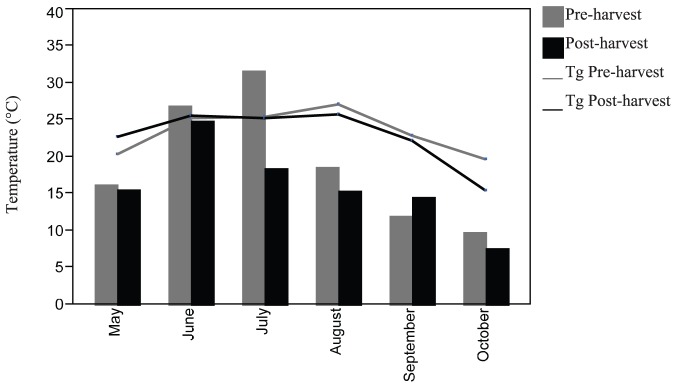
Pre vs. Post Movements. Average steplength (m/day) moved by eastern box turtles each month for both harvest periods (pre-harvest [2007–08] and post-harvest [2009–10]; bars). The average ground temperatures (Tg; °C) recorded at turtle location each harvest period are also plotted (lines).

Steplength decreased from pre-harvest to post-harvest (*F*_1, 66.2_ = 33.96, *p*<0.001) but there were no differences (all *p*-values>0.13) by sex, management class, or any combination of the three. The percent of steplengths that equaled zero (the percent of time the animals did not change position between GPS locations) was 1.83% pre-harvest and 0.86% post-harvest, meaning the animals moved more often post-harvest. Steplength was positively and significantly correlated with ground temperature (*R* ^2^ = 0.16, *p*<0.001; Figure 2a & b). Thermal optimum was found at 22–24°C pre-harvest (Figure 2c) and 24–26°C post-harvest (Figure 2d) despite the fact that ground temperatures were higher pre-harvest (mean = 24.5°C) than post-harvest (mean = 22.7°C; *F*_1, 7315_ = 140.8, *p*<0.001). Average steplength during the pre-harvest period was 22.08 meters (SE = 1.21) and 15.40 meters (SE = 0.88) during the post-harvest period, with the height of activity varying by month (Figure 3).

### Thermal habitats

**Figure 4:**
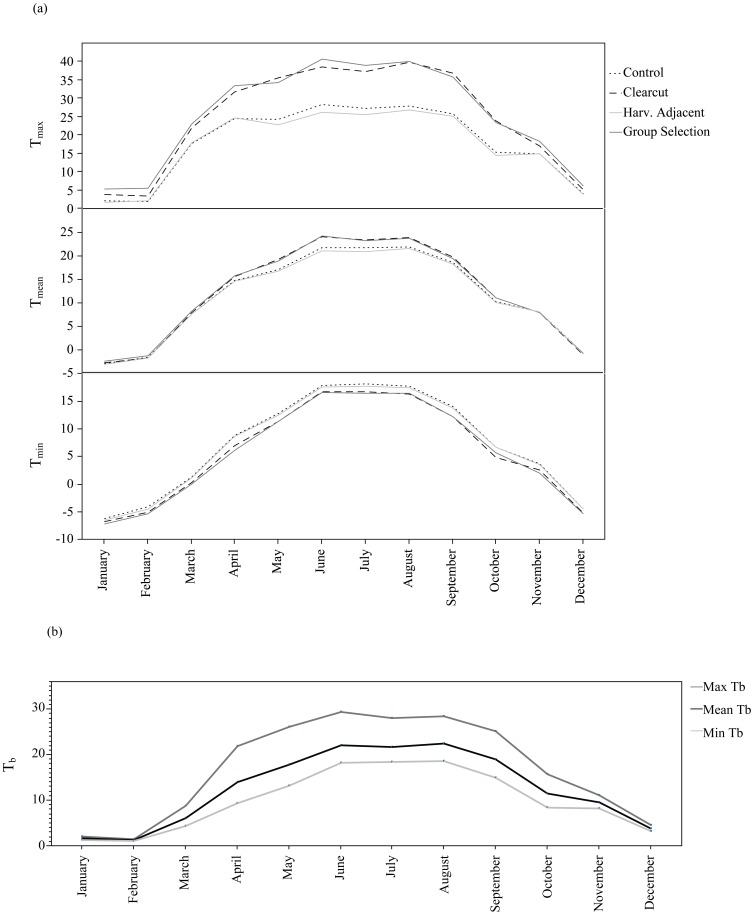
Habitat and Animal Temperature Ranges. Mean monthly temperature maxima (Tmax), mean (Tmean), and minima (Tmin) over two years (2009–2010) by habitat type (clearcut openings, group selection openings, harvest-adjacent forest (Harv. Adjacent), and forested control) (a). Maxima, means, and minima monthly eastern box turtle body temperatures (Tb) for the same period (b).

We processed 388,974 environmental temperatures from 36 locations in four habitat types (clearcut opening, group selection opening, harvest-adjacent forest, and forested control) between May 2009 and October 2010. Available temperatures differed at each level (T_max_, T_mean_, and T_min_) for each habitat type, month, and habitat by month interaction. The interaction term for T_min_ was the only non-significant effect (*F*_33, 376.6_ = 0.959, *p* = 0.54) in the model. The strength of the effects varied by month, with the harvest habitat types (clearcut and group selection openings) more similar to one another and forested habitat types (harvest-adjacent forest and forested controls) more similar (Table 2). Habitat type affected T_max_ more strongly than others. Explicitly, the range of temperatures for T_max_ was broader between habitats than for T_min_ or T_mean_ especially during the active period (Figure 4a). Between March and October, T_max_ in both harvest habitat types were significantly warmer (>10°C) than forested habitats (forests T_max_ = 24.57°C, SE = 0.73; harvest T_max_ = 34.43°C, SE = 0.80; *F*_1, 40.25_ = 83.56, *p*<0.001). This difference was most extreme in August when the T_max_ in harvest areas averaged 39.98°C (SE = 0.99) while it was nearly 13°C cooler in forested areas at 27.49°C (SE = 0.88). In contrast, T_min_ and T_mean_ differences remained within 3°C between habitat types, but usually less than 2°C for these months.

**Table 2:** Least Squares Means (LS Mean) Tukey-Kramer post-hoc pairwise comparisons connecting letters report of monthly environmental temperatures (Tmin, Tmax, Tmean) during 2009–2010 within four habitat types (clearcut openings, group selection openings, harvest-adjacent forest, and forested control).

Level	Habitat Type*			LS Mean
T_mean_	GroupSelection	A		12.4068993
	Clearcut	A		12.3435027
	Control		B	11.4761091
	Harv.Adjacent		B	11.1620106
T_max_	GroupSelection	A		25.3953822
	Clearcut	A		24.6201529
	Control		B	17.7994578
	Harv.Adjacent		B	17.2141375
T_min_	Control	A		7.302016
	Harv.Adjacent	A		7.05775033
	Clearcut		B	5.99306883
	GroupSelection		B	5.84889568

* Habitat types at each level not connected by the same letter are significantly different.

### Local-scale effects

We recorded and processed 494,548 body temperatures among 50 turtles between May 2009 and October 2010. The maximum, mean, and minimum T_b_ varied by month (Figure 4b). T_b_ was highly correlated with T_g_ (*R* ^2^ = 0.71, *p*<0.001). Behavioral categories were correlated with T_b_ over the post-harvest period, but explained very little of the variation (*R* ^2^ = 0.08, *p*<0.001). Post-hoc analysis revealed significant T_b_ differences in basking, walking, resting, and being underground behaviors. Behaviors associated with higher T_b_ (24–27°C) included basking and mating. Behaviors generally associated with lower T_b_ (22–23C°) included resting, inverted, walking, and eating, but post-hoc analysis revealed that these were not significantly different than mating. When T_b_ decreased to an average of 13.8°C, the animals were generally buried underground (near the hibernation season).

We found no significant difference in number of animal locations between the harvest periods within harvest-polygons. While the pre-harvest Euclidian distances within the designated harvest boundaries and their edges did not differ from 2007 to 2008, the averages were significantly different from post-harvest Euclidian distances in each polygon (*F*_1, 516.5_ = 32.45, *p*<0.001). Inside the harvest boundaries, post-harvest Euclidian distances were shorter (11.26 m, SE = 1.66) compared to pre-harvest Euclidian distances of 22.91 m (SE = 2.83). A similar trend was found within edge polygons where post-harvest Euclidian distances (14.45 m, SE = 1.27) were smaller than pre-harvest (23.60 m, SE = 2.10).

**Figure 5:**
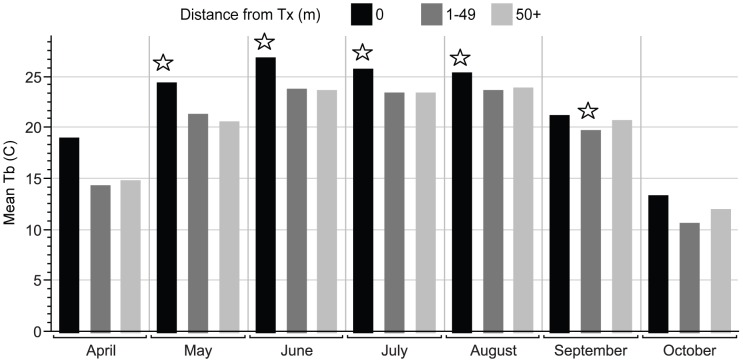
Harvest Proximity Temperatures. Mean eastern box turtle body temperatures (Tb) in degrees Celsius (C) with relation to timber harvest proximity over the active season months for post-harvest years (2009–10 combined). Starred bars represent significantly different mean temperatures during that month.

Body temperatures did not vary among management classes (*F*_2, 40.72_ = 1.624, *p* = 0.21) but were different among months (*F*_6, 294.7_ = 1087.334, *p*<0.001; Figure 5). However, animals found within the harvest openings maintained 9% higher T_b_ overall than those found in the forest/harvest edge or forest interior (*F*_2, 73.24_ = 8.135, *p*<0.001). Body temperatures within 50 meters of the harvest edges were lower (21.72°C, SE = 0.35) than farther inside the forest (22.22°C, SE = 0.21) and harvests (23.91°C, SE = 0.44).

## Discussion

Recent literature has shown that timber harvesting can have both positive and negative effects on forest dwelling species. Here we investigated the effect of various harvest openings on an ectotherm, the eastern box turtle. Using an experimental design and a variety of approaches, we demonstrate that in a relatively contiguous forested landscape, timber harvests have little effect on the short-term annual behavior of box turtles. However, we did detect a behavioral effect at the local scale where available microenvironmental temperatures were altered. We also offer further evidence that there is much variation in the annual behavior and home ranges of *T. c. carolina* that should be considered when establishing management strategies for forests and this species.

### Landscape-scale effects – home ranges and thermal ecology

Ectotherms, such as box turtles, will preferentially use certain types of available habitats for thermoregulation, nesting, and aestivation [50], [51]. Home range size likely depends on the quality of available food and other resources within the habitat [28]. Annual MCP home ranges for our adult *T. c. carolina* ranged from 0.47 and 187.67 hectares, the upper extreme being much larger than reports from any other study on this species. Indeed, our average annual home range estimate of 7.45 ha is more than 33% larger than any other published estimates to date (Table 3; [7], [45], [52]–[58]). It should be noted that there is a large variance in home range estimates across studies, which is likely associated with study duration, size, and monitoring method. The most likely explanation for the large home range size reported here is that our study was conducted within an expansive, relatively contiguous, and undisturbed habitat. Iglay et al. [59] found that turtles in fragmented habitats moved less often than those in contiguous habitats. To this end, many previous studies were conducted within relatively small and fragmented habitats that likely physically limited home ranges (Table 3).

**Table 3:** Published studies involving home range estimates from native populations of *T. carolina.*

Authors [Reference]	# of Animals (# of loc/turtle)	Duration of study	Method	Home Range Size Estimate	Location (study size)
Nichols [53]	12 (14)	20 yrs	Mark-recapture	120–200 m diam.	Long Island, NY
Stickel [84]	55 (3+)	3 yrs	Mark-recapture & thread trailing	100 m diameter	Maryland (11 ha)
Dolbeer [54]	‘many’ of 270 marked	1 yr	Mark-recapture	76.2 m diam.	Tennessee (8.9 ha)
Schwartz & Schwartz [50]	239 (4–18)	8 yrs	Dog capture-recapture & Radiotelemetry	1.9 ha ave. area;(1.2–10.2 ha)	Missouri (22 ha)
Madden [51]	23 (85)	4 yrs	Radiotelemetry	♀ 373 m diam.;♂ 284 m diam.;2.12 ha ave. area	New York
Strang [55]	8 (3+)	3 yrs	Thread trailing	167 m diam.	Pennsylvania (29 ha)
Schwartz & Schwartz [44]	37 (11–44)	19 yrs	Dog capture-recapture & Radiotelemetry	5.2 ha ave. area;(0.6–10.7 ha)	Missouri (22 ha)
Bayless [7]	6 (10+)	56 days over 2 yrs	Radiotelemetry & thread trailing	213 m diam.;1.25 ha	Virginia (49.4 ha)
Williams & Parker [56]	35 (3+)	26 yrs	Mark-recapture	♀ 176 m diam.;♂ 171 m diam.	Indiana (72.9 ha)
Hallgren -Scaffidi [57]	11 (3+)	2 yrs	Mark-recapture & thread trailing	97 m diam.;0.2 ha area	Maryland (11.3 ha)
Stickel [45]	103 (3+)	37 yrs	Mark-recapture	145 m diam.;♀ 1.13 ha area;♂ 1.2 ha area	Maryland (11 ha)
Quinn [58]	14 (av. 62)	1 yr	Radiotelemetry	♀ 4.0 ha area;♂ 6.7 ha area;4.97 ha ave. area	Connecticut
Current Study	50 (av. 34–70/yr)	4 yrs	Radiotelemetry	♀ 5.55 ha area;♂ 9.14 ha area;7.45 ha 4-yr ave.	Indiana (35,000 ha)

In this study, we found no differences in either annual or biennial home ranges across the landscape in association with any of the three management classes (clearcut opening, group selection opening, or control). This lack of variation was likely due to the fact that the actual timber harvest openings were relatively small (0.15–4.43 ha) in relation to *T. c. carolina* home range size and the surrounding contiguous forested habitat. Forest species often develop different strategies to cope with habitat perturbations. Some species expand their home ranges in response to forest fragmentation [60] while others inhabit territories that contain only small percentages of preferred habitat [61]. Still other species may gravitate toward mixed-composition habitat [62]. In the current study, the percent of animal locations within harvest edges did not change from pre- to post-harvest, suggesting that no such gravitation occurred. However, the movement parameters we investigated suggested that animals did alter their behavior while in proximity to harvest boundaries.

In pre-harvest years, animals tended to move longer distances (i.e., longer steplengths) than post-harvest years. However, the percent of steplengths that were zero were higher pre-harvest (1.83% vs. 0.86%). This suggests that although the animals moved shorter distances and maintained generally smaller home ranges after the harvests were implemented, they moved more often. These increased short-range movements may be the result of changes in resources. In this altered habitat, animals may need to move frequently for new foraging opportunities as seen with many small mammal and bird species [60], [63]. Shorter movements may be a result of downed slash acting as physical barriers or severe climatic conditions (i.e., drought). While it was evident that the animals did reduce movements during drought years, the cumulative effect on our results is minimal because the animals experienced drought years during pre-harvest 2007 and post-harvest 2010. Alternatively, behavioral thermoregulation may explain why the animals regularly moved but remained nearer to the same locations post-harvest.

Studies of fine-scale temperatures over broad spatial expanses are rare, despite the fact that temperature is an important factor in the location and activity of species [16]. A primary effect of the alteration of landscapes is the change in the microclimate of available habitats [64]. We measured these changes temporally across the landscape using temperature dataloggers. Although there was annual variation in ambient temperatures, the microclimatic conditions varied significantly between harvest and forested habitats. The most pronounced period occurred between May and September for T_max_ when differences were often greater than 10°C. These extreme summer temperatures found within harvest areas potentially exclude many plant and animal species. For example, variation in microclimates has been shown to affect the germination of emergent herbaceous and woody species [65]. During periods of highest temperatures, T_max_ within harvest areas was often observed to be near the maximum thermal tolerance for most ectotherms (43°C) effectively reducing the suitability of these areas for *T. carolina* (34.2°C; [66]) and other herpetofauna [67]–[70]. Although the current study examines a subset of factors affected by timber harvests, the advantage of this approach is the resulting detailed data of mechanisms underlying landscape effects [63]. Our results suggest that population-level responses to small-scale timber harvests (which are typical for the Midwestern U.S.) are minimal.

### Local-scale effects – movement and edge effects

Ecotones (either natural or man-made) will influence animal activity differently as surface temperature, air temperature, and canopy cover varies across the landscape [55], [71]. Ecotones at the harvest edges may provide cover by fallen logs and downed treetops, increased concentration and variety of forage (soft mast plants and invertebrates), and may facilitate behavioral thermoregulation by providing basking sites. Although we found no significant difference in the relative number of animal locations within the boundary or edges of the harvest areas, we did find differences in the movement parameters that suggest the animals use these areas differently. Prior to the harvests, the animals made longer, scattered movements across would-be harvest areas. Once the harvests were implemented, the movements (Euclidian distances) across the harvests shortened and were concentrated along the edges of the harvests (within edge polygons). Directed movements, although varied, often would alternate from the forest to the harvest edge, and frequently would cross project logging roads to do so. Studies on various turtle species have determined that roads bisecting animal routes were positively correlated with male biased sex ratios [27], [72]–[75], population declines [76], and expanded home range sizes [77]. In this study, two of the sites were bordered by public roads and all sites were adjacent to logging roads, however we did not analyze correlations of the roads to movements or home ranges.

Anthropogenic effects extend beyond the physical boundary of disturbance. In a broader definition of habitat, thermal microclimates limit the use of certain areas both seasonally and spatially. Analyses of the variables that affect ambient temperatures on a microclimate scale will aide in the understanding of habitat requirements of ectotherms [16]. In this study, the animals found inside the harvest areas maintained higher active season body temperatures than those outside the harvests by 10.13%. As expected, basking behavior correlated with higher temperatures. Forested sites located near roads or open areas such as timber harvests, are found to be generally warmer than those further away [16]. However, T_b_ at timber harvest edges were the lowest during the active period, even lower than in the adjacent forested habitat suggesting that the animals were moving between microhabitats for thermoregulation as seen in other taxa [78], [79]. The animals within our experimental openings were exposed to a wide range of temperatures. In a laboratory study, the specificity of T_b_ was investigated between *T. c. carolina* and *T. ornata* with the finding that *T. c. carolina* has less thermal specificity [39]. We routinely found the animals walking while inside the harvests and document that they do have the ability to behaviorally adjust to varying temperatures at a fine scale. These adjustments may play key roles in the physiological requirements of ectotherms throughout ontogeny and in various physical conditions (e.g., in reptiles, gravid females actively adjust to maintain higher body temperatures than males; [80].

Open spaces, such as clearcuts, may have less of an effect on larger-bodied species or those adapted to hot and dry conditions. Canopy cover directly influences light intensity, which is known to be a critical factor for many reptiles during activity periods [81]–[83]. On the other hand, many reptilian species such as small-bodied snakes are adapted to utilize leaf litter and are likely to be adversely affected by its removal with associated timber harvests [83]. During the active season, *T. c. carolina* use leaf litter to create ‘forms’ as cover [84]. *T. c. carolina* will use these forms throughout the active period as refuge from the heat, cold, rain, or disturbance [28]. In addition to cover, leaf litter serves as habitat for prey (such as snails, worms, and mushrooms) of box turtles. Immediately following implementation of harvests, leaf litter is degraded, blown from these areas, and often leaves large patches of unsuitable bare ground [85]. Studies have found that the increased soil temperatures and reduced leaf-litter cover (which can take decades to return pre-harvest conditions) in previously cut areas exclude many amphibian species [86], [87]. We found that short term effects such as the loss of leaf litter did not cause the animals to abandon the area, but rather continue to use it in a different way (such as for thermoregulation).

Merely reporting species declines without determining their mechanistic causes leaves conservation planners with little recourse. To date, no studies have monitored the response of an ectotherm's movement parameters prior to and after discrete anthropogenic disturbance such as timber harvests. The present study has yielded detailed data on habitat use and spatial ecology of an ectotherm in a managed forest, but has much broader implications on multiple forest-dwelling species in a changing climate. In our study, the timber harvest openings were generally small (<5 ha) and were contained in a relatively contiguous and much larger forest matrix. Our results indicate that in a relatively contiguous forested landscape, small-scale timber harvests have minimal effects on the short-term behavior of these ectotherms. However, temperature fluctuations as seen in the current study affect seasonal available habitat for forest-dwelling animals, especially for those with limited dispersal and thermoregulatory capabilities. Microclimates within harvested areas can exclude animals, but they also may create some desired ecotonal habitat. Considerations of habitat requirements and contiguity of surrounding refugia habitat and species ability to recover should be thoroughly considered before timber harvest sizes are determined. These factors are of particular interest when dealing with long-lived species of conservation concern amid a changing climate.

## Supporting Information

Table S1 **Four-year home range summary.** Summary of turtle annual home ranges at all nine study sites from 2007–2010. (PDF). Legend: Summary of the eastern box turtle annual home ranges at nine study sites in south-central Indiana from 2007–10. Year, sex, management class (Mngmt Class), number in group (*n*), and median, mean, and standard errors of annual home range (100% Minimum Convex Polygon; MCP) in hectares (ha). For 2007–08, the management class represents the assigned harvest type prior to harvest implementation.
(PDF)
