# Supplementary material for: Short-Term Forest Management Effects on a Long-Lived Ectotherm
Source: PLoS One. 2012 Jul 6;7(7):e40473. doi: 10.1371/journal.pone.0040473 (PMC3391286; doi:10.1371/journal.pone.0040473)
Supplement: Table S1 — Four-year home range summary. Summary of turtle annual home ranges at all nine study sites from 2007–2010. (PDF). Legend: Summary of the eastern box turtle annual home ranges at nine study sites in south-central Indiana from 2007–10. Year, sex, management class (Mngmt Class), number in group (n), and median, mean, and standard errors of annual home range (100% Minimum Convex Polygon; MCP) in hectares (ha). For 2007–08, the management class represents the assigned harvest type prior to harvest implementation. (PDF) [file pone.0040473.s001.pdf]

Table S1. Four-year home range summary. Summary of the eastern box turtle annual home ranges at nine study sites in south-central Indiana from 2007–10. Year, sex, management class (Mngmt Class), number in group (*n*), and median, mean, and standard errors of annual home range (100% Minimum Convex Polygon; MCP) in hectares (ha). For 2007–08, the management class represents the assigned harvest type prior to harvest implementation.

Year	Sex	MngmtClass	*n*	Median Area	Mean Area	SE
2007	F	Clearcut	4	4.76	16.44	12.747
		Control	4	1.63	1.62	0.274
		GroupSelection	2	2.26	2.26	0.360
	M	Clearcut	4	1.59	2.03	0.540
		Control	4	1.90	34.02	32.289
		GroupSelection	5	2.89	3.58	1.032
2008	F	Clearcut	5	3.04	3.04	0.568
		Control	3	4.77	5.40	2.463
		GroupSelection	2	9.28	9.28	7.185
	M	Clearcut	5	1.97	3.84	1.469
		Control	4	4.17	49.50	46.064
		GroupSelection	7	1.20	4.31	2.966
2009	F	Clearcut	7	2.16	9.77	5.835
		Control	7	6.57	6.69	1.712
		GroupSelection	7	2.11	3.94	2.025
	M	Clearcut	6	2.94	3.01	0.664
		Control	6	2.48	17.57	15.510
		GroupSelection	8	1.85	1.99	0.405
2010	F	Clearcut	7	1.64	2.11	0.641
		Control	7	2.50	5.38	2.369
		GroupSelection	8	2.07	3.48	1.358
	M	Clearcut	7	2.48	9.02	6.155
		Control	7	2.20	2.97	1.041
		GroupSelection	8	1.71	2.05	0.345
